# Comparison of Language and Memory Lateralization by Functional MRI and Wada Test in Epilepsy

**Published:** 2021-04-16

**Authors:** Natalie N. Htet, Ricardo Pizarro, Veena A. Nair, Daniel Y. Chu, Timothy Meier, Evelyn Tunnell, Paul Rutecki, Bruce Hermann, Elizabeth M. Meyerand, Vivek Prabhakaran

**Affiliations:** 1 Department of Radiology, University of Wisconsin School of Medicine and Public Health, USA; 2 Department of Biomedical Engineering, University of Wisconsin Madison, USA; 3 Department of Neurology, University of Wisconsin School of Medicine and Public Health, USA; 4 Department of Medical Physics, University of Wisconsin School of Medicine and Public Health, USA

**Keywords:** fMRI, language, memory, epilepsy, Wada test

## Abstract

The intracarotid sodium amobarbital procedure (ISAP or Wada test) lateralizes cerebral functions to the cerebral hemispheres preoperatively. Functional magnetic resonance imaging (fMRI) is increasingly used to characterize preoperative language and memory lateralization. In this study, concordance of fMRI with Wada was examined in patients with medically intractable seizures. The relationship of the distance between the epileptogenic focus to functional activation area with patients’ post-operative deficits in language was also analyzed. 27 epilepsy patients with preoperative fMRI and Wada data were analyzed using established fMRI paradigms for language and memory. Activation of Broca’s and Wernicke’s areas were measured in three dimensions. Language and memory lateralization were determined, and standard neuropsychiatry Wada test procedures were used for comparison. The shortest distance between a language area to the border of surgical focus (LAD) was also measured and compared with postoperative language deficits. Our study found that concordance between fMRI and Wada testing was 0.41 (Kappa’s ‘fair to good’ concordance) for language dominance and 0.1 (Kappa’s ‘poor’ concordance) for memory. No significant correlation was found between LAD and post-op language deficit (*p*=0.439). A correlation was found between LAD and post-op memory deficit (*p*=0.049; the further distance from surgical lesion to language area is associated with less post-operative memory loss). Females demonstrated significantly increased postoperative seizure improvement (Fisher’s p-value=0.0296; female=8; male=6). A significant association between handedness (right-handed subjects) and postoperative seizure improvement was found (*p*=0.02) as well as a significant trend for interaction of gender and handedness on postoperative seizure improvement (*p*=0.09). Overall, our results demonstrate fMRI as a useful preoperative adjunct to Wada testing for language lateralization in patients with medically intractable seizures.

## Introduction

Surgical treatment of epilepsy is commonly considered for patients with medically intractable seizures [1]. Especially in anteromesial temporal lobe resection, the benefits of surgery outweigh the risks of continued trials of treatments with anti-epileptic drugs [2]. In temporal lobectomy, 66% of patients remained seizure free at 5-year follow-up [3]. However, verbal memory deficits are observed in patients with left hemispheric resection post-operatively [4], and language impairment is a concern due to the proximity of mesial temporal lobe to language regions. In order to minimize post-operative deficits, assessment of language and memory lateralization is essential for the neurosurgeon’s pre-operative planning.

The gold-standard for pre-surgical determination of language and memory dominance thus far has been the Wada test or intracarotid amobarbital procedure [5,6]. However, Wada is an invasive, expensive procedure limited to lateralization and not functional localization [6,7]. It is also an invasive study, and potentially harmful, with a complication rate of up to 11%, including stroke and carotid dissection in up to 1% [8]. Although these issues exist, Wada is still clinically being used for language and memory lateralization.

Functional magnetic resonance imaging (fMRI) has been increasingly studied as a non-invasive alternative to the Wada test. FMRI offers a safer, more cost-effective alternative with the advantage of complete brain coverage [7]. Several studies have found concordance percentages in the range of 75–95% between fMRI and the Wada test, and presently, solutions to increase its reliability are being explored [9–13]. There is also data that shows enhanced benefits of using fMRI to predict post-operative deficits in comparison to the Wada test [14].

In this study, we compare lateralization for language and memory as determined by the Wada test and fMRI in epilepsy patients who have undergone both testing in our hospital. We present our results in light of those reported in the literature and discuss important issues that affect the use of fMRI in determining hemisphere dominance that should be considered by neurosurgeons. Previous work has also demonstrated that the use of pre-operative fMRI and Wada in epilepsy patients can be helpful in predicting post-operative memory outcomes [15]. Therefore, we also explored whether measures derived from fMRI and Wada, the distance from lesion to fMRI activation, as well as lateralization as determined by Wada and fMRI, are predictive of post-operative deficits in language and memory. Given the evidence that there may be a susceptibility by gender to the development of epilepsy and its subtypes, we also explored gender differences in our sample, with particular focus on seizure remission and medication use in these patients [16]. Finally, we also investigate and discuss additional factors that are associated with seizure remission, including language and memory lateralization as determined by Wada and fMRI, duration of epilepsy symptoms, presence of mesial temporal sclerosis on MRI, presence of pre-operative MRI abnormalities, and correlation of EEG with structural abnormalities detected on MRI, handedness, in addition to gender.

## Materials and Methods

### Study subjects

Subjects were selected from a database of 522 patients who underwent fMRI as a part of pre-surgical planning at the University of Wisconsin, Madison between June 1999 and Sept 2011. Twenty-seven patients with a diagnosis of medically intractable epilepsy and who underwent both Wada and fMRI for language and/or memory mapping were included in this study. One patient was discovered to have a brain tumor; two patients had a vascular malformation. The 24 patients were discussed at the weekly Epilepsy conference attended by neurosurgeons, neurologists, neuropsychologists, and neuroradiologists. Surgery was offered to patients when the meeting attendees were in agreement about the benefits of surgery. Six patients did not continue with surgery, either due to the risks of surgery or the high chance of language or memory deficit. Eighteen patients had anterior temporal lobectomy. The study was carried out under the approval of the Institutional Review Board of the University of Wisconsin Madison.

Patients followed up with their respective neurosurgeons and neurologists at one month, three to six months, and yearly after surgery. Postoperative language or memory deficits and seizure recurrences, as noted by neurosurgeons or neurologists in medical records, were included in the analysis. Due to the longitudinal nature of study, patients had follow-ups ranging from 3 months to 3 years. Out of 27 patients included in this study, 16 were female (15 right-handed and 1 left-handed) and 11 were male (6 right-handed, 4 left-handed, and 1 ambidextrous).

### Wada test

The Wada test was performed on patients to determine language and memory dominance. For each patient, each hemisphere was temporally anesthetized via intracarotid injection of sodium amytal. The patient was then asked to name target items, and spontaneous expressive speech and receptive language comprehension were assessed. Hemispheric language dominance was determined from the deficit associated with each hemisphere under anesthesia [5], and hemisphere dominance was categorized as left hemisphere, right hemisphere, or bilaterally dominant.

To determine hemisphere dominance for memory, patients were presented with eight to-be-remembered items approximately 160 seconds following administration of sodium amytal. Memory was then assessed 10 minutes after the injection, and the number of correctly remembered items was recorded for each patient. Hemisphere dominance was categorized as being left, right, or bilateral based on the patient’s performance. The Wada test performed at our institution is in line with the methods reported since 1960 and used for decades clinically by neuropsychologists and neurosurgeons [5].

### Functional MRI

#### Paradigms for language and memory tasks

The language and memory paradigms used to assess patients are described in more detail by Moritz and Haughton [17]. Briefly, Broca’s area activation, which is associated with speech production, was elicited with two word-generation fMRI tasks: 1) alternating 20-second blocks of antonym word generation versus rest, and 2) alternating 20-second blocks of letter-word-generation versus rest. Wernicke’s area activation, which is associated with receptive language, was identified with alternating 20-second blocks of text-reading-versus-symbols. In this task, the patient silently read a short paragraph in the text-reading block. During the control or symbols block, the patient was shown a paragraph of symbols and asked to find specific symbols. The symbols block controlled for eye movements during reading, which is designed to discriminate visual and eye movement–related activity from the true language areas.

Activation associated with memory was determined using two scene encoding tasks. Pictures of either New York City or Egypt and abstract images of square tiles appeared in random order at a rate of one image per four seconds. Patients were asked to memorize the photographs of the scenes but not the abstract images. Immediately following the scan, a series of photographs were shown, some of which were seen previously and some which were not and patient was asked to identify by responding with yes or no for each photograph.

#### FMRI acquisition and processing

Imaging was performed and used either a 1.5T or 3T commercial MR imaging scanner (Sigma GE Healthcare, Waukesha, Wisconsin). Blood oxygenation level-dependent (BOLD)-weighted single-shot EPIs were obtained at periodic repetition time intervals (TR) for each patient during task performance. Imaging parameters were: FOV=24cm; matrix-size=64 × 64; TR=2000ms; TE=40ms (for 1.5T) or 27ms (for 3T); flip-angle=85° (for 1.5T) or 75° (for 3T); 6mm coronal plane sections (for 1.5T) or 5mm axial plane sections (for 3T). Spatial coverage was sufficient to provide mapping of the entire cortex. The number of images and the length of imaging varied with the paradigm. Imaging duration ranged from 3 to 5 minutes. Additional high-resolution anatomic scans, including 3D volumetric T1 and T2 weighted sequences were acquired as part of the preoperative assessment. All post-processing was performed using AFNI [18]. First, all data was spatially smoothed using a 6mm FWHM Gaussian kernel. Second, temporal alignment was performed to account for differences in temporal acquisition at each slice location within the TR of 2 seconds. Finally, all data was spatially co-registered: first to the middle volume of the fMRI run then to the structural image acquired in the same session. Activation was determined by cross-correlation of the EPI time-course signal intensity at each voxel with a generalized least-squares fitting algorithm to a smoothed and temporally delayed boxcar reference function modeling the hemodynamic response. This correlation resulted in a voxel-wise t statistic that was used to threshold images and to optimize visualization of language or memory areas. The resulting activation maps were overlaid onto the co-registered anatomic brain volumes.

The thresholding level was determined by an experienced technologist aiming to maximize visibility of FMRI task activation while minimizing artifact contamination. This meant adjusting the threshold to minimize spurious voxels that were considered artifacts (e.g., due to head motion). It also involved adjusting the threshold to highlight the expected responses (e.g., Broca’s area or medial temporal regions) at a level that displayed a typical supra-threshold extent. This was subjectively adjusted to localize a particular gyrus or region, which represented a statistical probability of greatest confidence as indicated by the t statistical overlay. Often, a compromise threshold was applied to balance the need to highlight an expected response with the concern of minimizing artifacts. For example, in a dataset that exhibited significant task-correlated head motion, it may not have been possible to minimize the artifacts while still retaining sufficient sensitivity to the presumed task-related responses. The task-related response magnitudes were also dependent on factors such as the patient’s ability to perform a particular fMRI task. Thus, the threshold was varied for each individual fMRI scan on the basis of the quality of the data to maximize the pre-operative utility of the scan for each individual patient.

#### Language lateralization

**Figure 1.**
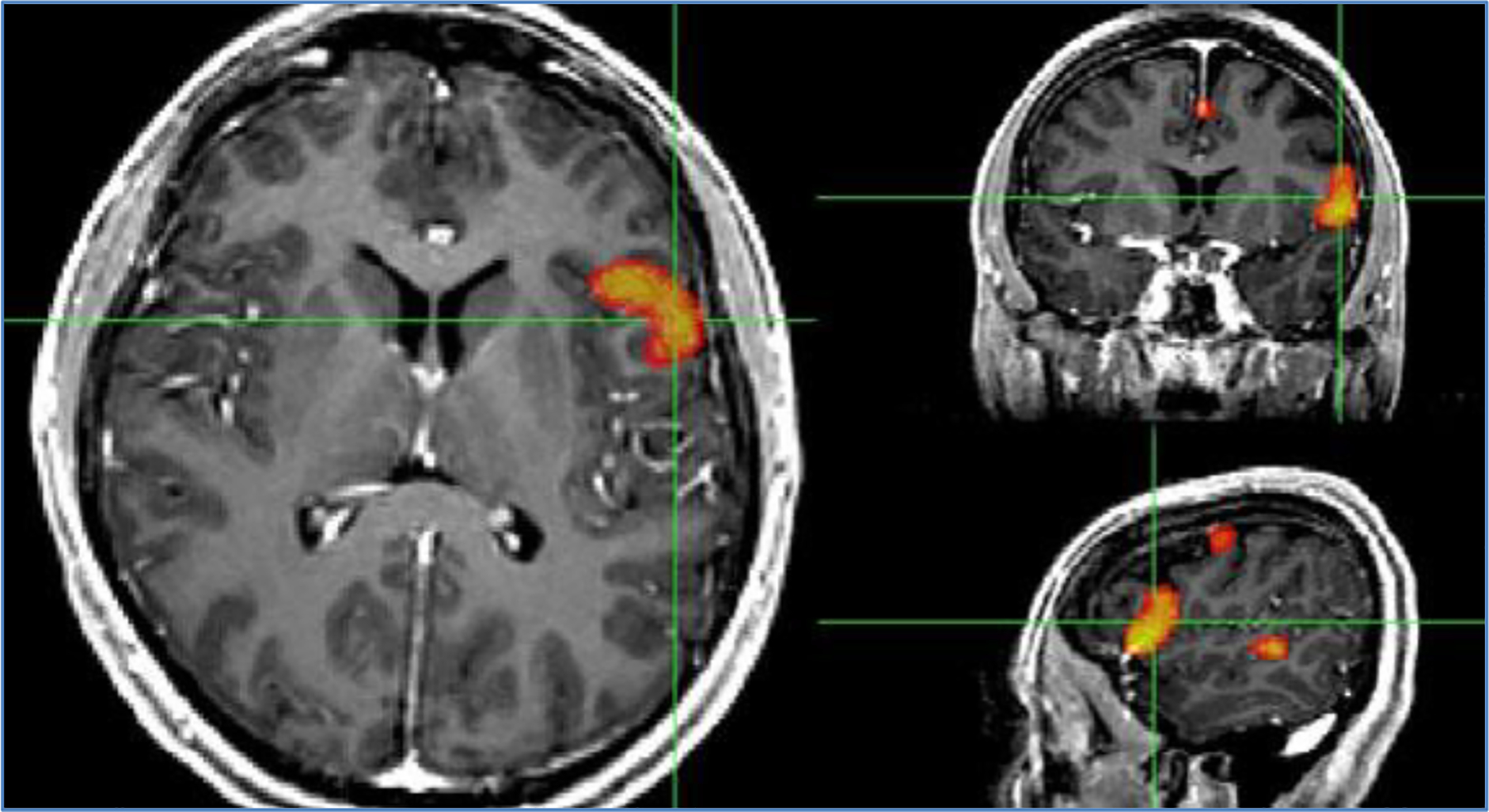
Patient showing left lateralized language activation in the left inferior/middle frontal regions and left posterior/superior temporal regions

Using the FMRI activation maps, the amount of activation was computed in the two regions known to correspond to language function (Figure 1): Broca’s or left inferior frontal region and Wernicke’s area or left posterior superior temporal region. By simultaneously looking at the activation maps and the structural image in PACS, we measured the maximum width (x_max_) and maximum height (y_max_) of the activation by scrolling through each axial slice. In addition, the activation thickness (z_max_) was computed by counting the number of axial slices where activation was present and multiplying by the slice thickness [19]. Activation for each region was calculated as:

eq ① $$\text{Activation }\left({\text{mm}}^{3}\right)=\frac{\left(x_{\text{max}}\times y_{\text{max}}\times z_{\text{max}}\right)}{2}$$

The activation was computed for both left and right hemispheres and the same method used for the activation in Wernicke’s area. A threshold-dependent Lateralization Index (LI) was calculated for language areas by comparing the amount of activation in the Left and Right hemispheres:

eq ② $$LI=\frac{1}{2}\times \left[\frac{\left(\text{ Left }-\text{ Right }\right)}{\left(\text{ Left }+\text{ Right }\right)}\right]$$

Left is the amount of activation in the left hemisphere and Right is the amount of activation in the right hemisphere as computed above in equation 1. LI indicates the degree of left or right hemispheric dominance for a subject. LI>0.2 was categorized as left hemisphere dominance, LI between −0.2 and 0.2 was categorized as bilateral dominance, and LI<−0.2 was categorized as right hemisphere dominance.

#### Memory

For pre-operative determination of memory dominance, the clinical fMRI report prepared by the experienced fMRI technologist and finalized by a Neuroradiologist provided interpreted activation maps for the memory tasks and determined memory lateralization as being left, right, or bilaterally dominant. This classification was used for these analyses.

### Statistical analysis

#### Wada test vs. fMRI

Wada and fMRI lateralization for language were compared in 26 patients; one patient did not complete fMRI language tasks. Wada memory test results were compared with fMRI memory results in 16 subjects; 11 patients did not have conclusive memory studies. For both memory and language tasks, patients were classified as being left dominant, or either right or bilaterally dominant. Cohen’s Kappa coefficient of concordance was computed to assess agreement between the methods of determining lateralization.

#### Association between pre-operative factors and post-operative language or memory deficits

We investigated whether the distance from epilepsy focus to the closet language area, either Wernicke’s or Broca’s, was associated with post-operative outcomes in language or memory. This information can be useful for surgeons to pre-operatively predict the outcomes of language or memory function after surgery using fMRI and to plan their resection approach. This method has been previously validated in brain tumor patients undergoing pre-surgical functional mapping [12].

Out of the pool of epilepsy patients who underwent anterior temporal lobectomy, 15 patients had both a follow-up MRI scan after surgery and post-op visits with their respective neurologists or neurosurgeons up to 2 years. Using pre- and post-operative imaging, the seizure focus and site of surgery was identified. By comparing post-operative MR images with pre-operative functional MR images, the shortest distance between the language region to the border of surgical focus (lesion-activation distance; LAD) was measured. Based on the patients’ follow-up visits, reports of language or memory deficits and the length of deficits were recorded. These patients were classified as having no post-operative deficits, transient post-operative deficits (less than 6 months), or persistent post-operative deficits (greater than 6 months). The association between LAD and post-operative deficits was tested for using Fisher’s exact tests. Similarly, the association between post-operative deficits and lateralization as determined by Wada test, as well as fMRI, was also analyzed via Fisher’s exact tests.

#### Association of pre-operative factors and seizure remission

The patients’ seizure occurrences at their follow-up visits with neurologist or neurosurgeons at 1–3 months, 6 months, 1 year and 2 years were noted to identify factors associated with seizure recurrence. Out of all patients with anterior temporal lobectomy, 14 patients were included in the analysis. Habitual outbreaks up to 2 episodes due to medication non-compliance were not considered as seizure episodes, and auras were not considered as seizures. Subjects had varied lengths of follow-ups from the time of surgery. All 14 patients had one to three months post-operative visits. Only 11 patients had surgeries remote enough to allow for collection of two-year follow-up data.

Adjustments of seizure medication in regard to an increase or decrease in dose or number of seizure meds were noted. The association of seizure remission and multiple factors collected from medical records of patients was investigated using Fisher’s exact tests, independent t-test, and analysis of variance (ANOVA). These factors included lateralization as determined by Wada and fMRI, duration of epilepsy symptoms, presence of mesial temporal sclerosis on MRI, presence of pre-operative MRI abnormalities, correlation of EEG with structural abnormalities detected on MRI, handedness, and gender. For all statistical tests, *p*<0.05 was treated as statistically significant.

## Results

### Concordance between Wada test and fMRI

Each subject’s results for language and memory testing as determined by both Wada and fMRI were categorized as left or right/bilateral (Table 1A and Table 1B). Cohen’s kappa coefficient of concordance was computed to be 0.41 for language, with fMRI and Wada agreeing on 22 of the 26 patients. Using the guidelines for computing concordance of multinomial functions published by Fleiss [20], fMRI and Wada have “fair to good” concordance for language lateralization.

Wada and fMRI agreed on 14 of the 16 patients for memory lateralization. However, fMRI classified all patients as having right or bilateral dominance, while Wada classified 2 patients as having left hemisphere dominance. Therefore, Cohen’s kappa coefficient of concordance was 0.1 for memory lateralization. fMRI and Wada had “poor” concordance for memory lateralization.

### Predicting language or memory outcomes after surgery

Distance from surgical lesion to language center (LAD) was categorized into less than 1cm, 1cm–2cm, or greater than 2cm for 15 patients (Table 2A and Table 2B).

A statistically significant association was found between LAD and post-operative memory deficits (*p*=0.049). The longer LAD is associated with absence of memory deficit post-operatively. No significant association was found between LAD and post-operative language deficits (*p*=0.439). No association was found between language lateralization as determined by fMRI and post-operative language or memory deficits (*p*=0.60 for LI and language; *p*=0.11 for LI and memory). In addition, language lateralization determined by Wada has no association with post-operative language or memory deficits (*p*>0.1). No association was found between memory lateralization determined by Wada and post-operative language or memory deficits (*p*=0.272 for language deficits; *p*=0.51 for memory deficits).

### Preoperative indicators of seizure recurrence

In this study six variables were tested for association with seizure remission: duration of epilepsy symptoms, presence of mesial temporal sclerosis on MRI, presence of pre-operative MRI abnormalities, correlation of EEG with structural abnormalities detected on MRI, gender, and handedness. A significant association was found between gender and seizure remission at one to three months, six months, one year and two years follow-ups (*p*=0.02, independent t-test) with more women (8 out of 8) showing seizure remission than men (3 out of 6) (Table 3). An association between handedness and seizure remission was found (*p*=0.02). Right-handedness is associated with a better seizure remission. For seizure remission, the gender by handedness interaction shows a trend towards significance (*p*<0.09, ANOVA). The association between seizure remission and all other factors was not significant (*p*>0.1).

## Discussion

Seizure management in patients with epilepsy is difficult as there needs to be a balance between avoiding side effects of anti-epileptic medications and improving quality of life. Surgery to remove the epileptogenic cortex can offer a significant reduction of seizures in patients with medically intractable epilepsy. In order to minimize the risks of compromised language or memory function after surgery, pre-operative assessment is critical. For decades, hemispheric dominance has been assessed in patients with epilepsy using the invasive Wada test [5]. Functional MRI methods offer a non-invasive alternative to identify the hemispheric language and memory dominance prior to surgery, allowing pre-surgical risk determination for language or memory loss.

### Concordance between Wada and fMRI

#### Language

Our results demonstrate that fMRI is comparable to Wada testing for pre-operative assessment for language dominance, which is consistent with numerous previously reported studies [21–23]. In regard to language, fMRI and Wada had ‘fair to good’ concordance per Kappa statistic with 84.6% agreement. Several studies have reported high concordance rates in the range of 80–90% in 21 published studies as noted in the review papers by Binder et al. [14,24]. In a meta-analysis, the sensitivity and specificity of fMRI compared to Wada test was reported to be 84% and 88% [21]. Functional MRI offers a safer, more cost-effective alternative with the advantage of complete brain coverage [7]. Moving forward several studies have begun to explore for a more robust thresholding approach to increase the concordance rates for all patients [10,11,25].

#### Memory

Although we observed a ‘poor’ concordance per Kappa statistic between Wada and fMRI for lateralization of memory, fMRI and the Wada test did agree on 87% or 14 of the 16 subjects. However, the fact that fMRI did not classify any patients as having left lateralized memory, while the Wada test did, lowered the calculated concordance level. In our experience, the regions targeted by memory tasks, primarily the hippocampus, are often too small, making definitive characterizations of memory lateralization for memory using fMRI difficult. Likewise, others have reported that fMRI has been found to be individually variable and not reliable in terms of memory localization and lateralization, even in healthy volunteers [26]. This has been partly attributed to the reduced sensitivity of the BOLD effect in functional MRI in the hippocampus [24,27,28]. In addition, medial temporal lobe is functionally organized with various sub-regions (hippocampus, entorhinal and parahippocampal cortex) contributing to specific subprocesses of episodic memory [29]. In comparison studies between healthy subjects and patients with temporal lobe epilepsy, the memory lateralization index calculated based on fMRI memory-encoding paradigm yields concordance with Wada only in right-sided temporal lobe epilepsy [24]. Memory lateralization by fMRI is confounded by susceptibility artifacts especially in lateral and medial temporal lobe regions leading to poor signal-to noise ratio in these regions. It has been suggested that in-plane resolution less than 2 mm and thinner slices (<3mm) may improve SNR in the medial temporal lobe regions [30,31]. Large multicenter studies with improved scan parameters are needed to determine the potential of fMRI in pre-surgical evaluation of memory function in temporal lobe epilepsy, and to examine concordance of fMRI with Wada before fMRI can be routinely used in clinical practice [32].

### Postoperative outcomes

In terms of predicting outcome, we observed that neither Wada nor fMRI classification of hemisphere dominance was associated with post-operative deficits. Similarly, no association between LAD and post-operative language deficit was found. This could be a result of the limited number of patients in our study, but also an effect of inclusion of the Wada or FMRI test results in surgical decision making. Patients that had a low predicted probability for surgical deficits as assessed by Wada and FMRI testing were more likely to undergo surgery.

However, an association between LAD and post-operative memory deficits was significant. It is difficult to clarify specific memory deficits, such as naming deficit or verbal memory deficits, in our retrospective study. Sabsevitz et al. have reported that the temporal lobe fMRI LI can predict language decline assessed by the Boston Naming Test in patients undergoing anterior temporal lobectomy [33]. In addition, fMRI has been studied as a preoperative predictor of verbal episodic memory outcome in temporal lobe epilepsy [15]. However, there is wide variability in language protocols and patient populations, and a larger study is needed [34].

### Factors influencing seizure remission

Certainly, prevention of seizure remission is an expected goal for a majority of patients, and multiple factors have been reported to predict it. For example, temporal lobe resection, structural abnormalities found on preoperative MRI, the concordance between the location of ictal onset with electroencephalogram (EEG) interictal epileptiform discharges, IQ greater than 70, younger age, and shorter epilepsy duration were found to be associated with higher chances of seizure control after surgery [3,35–41]. One factor that is often neglected is the effect of gender. The only factor in our study that was significantly associated with seizure remission was gender; lateralization as determined by Wada and fMRI, mesial temporal sclerosis, pre-operative MRI abnormalities, EEG correlation with MRI abnormalities, and duration of epilepsy did not have statistically significant correlation with seizure remission in this study. It should be noted that handedness was also significantly associated with gender in our study subjects. A significant association between handedness and seizure remission was found as well as a significant trend for interaction of gender and handedness on postoperative seizure improvement. Besides handedness, gender groups have no significant difference in terms of age or type of surgery. Our study’s report of gender influence as well as handedness on seizure remission has not been reported in previous studies. Patients with epilepsy have atypical organization of the language function, and reorganization of language function in epilepsy patients has been known to occur [42]. Gender variations have been seen in cortical localization of naming as determined by stimulation mapping [43], although there are conflicting studies in regard to gender differences in reorganization of language [44]. Studies investigating gender differences in language lateralization, have reported conflicting results or no significant differences; for example, Ihnen et al. found no consistent gender differences in language processing [45], but Clements et al reported that men were more left lateralized during a phonological processing task, whereas females showed greater bilateral activity during the phonological task [46]. However, these reported differences in study results may vary as a function of task type and subject demographics. Notably, McGlone et al. found that men are more likely to become aphasic after a stroke than women [47]. This suggests that women tend to have a higher degree of brain plasticity than men while the general functional organization for language processing does not differ significantly, which could likely be due to hormonal influences [48]. The question of whether real gender differences in laterality exists is still an empirical one and whether resulting differences in fMRI activation maps influences surgeons’ aggressiveness in surgical resection needs further investigation. However, gender differences in brain plasticity for recovery from brain injury have been previously reported [49,50], indicating that gender differences need to be further investigated in a larger subject population.

In our study, more right-handed people than left-handed showed seizure remission. In a study with a large sample (N=327) cognitively healthy older adults, there is evidence that handedness is a significant predictor of changes in structural differences with aging; those who were left, particularly ‘mixed’ in handedness were more likely to have greater atrophy in hippocampus and amygdala [51]. Handedness is associated with brain atrophy which may lead to different levels of susceptibility to seizures and therefore may affect seizure remission.

## Limitations

The current study had a number of limitations. Language-related fMRI activation maps were statistically thresholded on an individual bases since the original intent of imaging was to provide patient-specific information for preoperative planning. Growing evidence suggests that the BOLD signal is sensitive to type of task as well as thresholding effects [52] and language lateralization could also vary as a function of these factors. Also, data reported here were collected on multiple scanners (1.T and 3.0T) depending on whether the patient was an in-patient or out-patient and this factor was not specifically taken into account in our study. The number of subjects in our study was modest which may have underpowered our statistical tests.

## Conclusion

This study provides results consistent with the literature that there is a high degree of concordance between fMRI and WADA for language lateralization, relatively less for memory lateralization. Additionally, similar to the previous studies that have shown significant association between LAD and postoperative deficit in other patient populations, an association was observed between LAD and postoperative memory deficit in the current study. This result needs further validation in a larger sample of epilepsy patients. Finally, we report an effect of gender and handedness in seizure remission with more females showing remission compared to males; we speculate on the protective effects of language bilaterality in females and right-handed subjects compared to left hemisphere dominance in males and left-handed subjects; however, these effects need to be replicated in a larger sample.

## Figures and Tables

**Table 1A:** Lateralization for language as predicted by fMRI and Wada

	fMRI Language			
**Wada Language**		**Left**	**Right or Bilateral**	**Kappa statistic**
	**Left**	20	2	0.41
	**Right or Bilateral**	2	2	

**Table 1B:** Lateralization for memory as predicted by fMRI and Wada

	fMRI Memory			
**Wada Memory**		**Left**	**Right or Bilateral**	**Kappa statistic**
	**Left**	0	2	0.1
	**Right or Bilateral**	0	14	

**Table 2A:** Correlation of LAD with post-operative language deficits

	LAD			
	Less than 1cm	1cm–2cm	Greater than 2cm	*p*-value
**No language deficit**	2	1	9	0.439
**Language deficit**	0	l	2	

**Table 2B:** Correlation of LAD with post-operative memory deficits

	LAD			
	Less than 1cm	1cm–2cm	Greater than 2cm	*p*-value
**No memory deficit**	2	l	9	0.049
**Memory deficit less than 6 months**	0	l	2	
**Memory deficit longer than 6 months**	0	0	1	

**Table 3:** Correlation of seizure remission with gender

	Female	Male	*p*-value
Patients with less than or equal to one seizure recurrence after surgery	8	3	0.02
Patients with greater than one seizure recurrence after surgery	0	3	
